# Obstetric and perinatal outcomes in female survivors of childhood or adolescent cancer: Protocol of a meta-analysis of population-based cohort studies

**DOI:** 10.1371/journal.pone.0273561

**Published:** 2022-09-02

**Authors:** Fang Deng, Xiuan Gao, Limian Xu, Weijie Li, Zubing Mei, Caijun Xie

**Affiliations:** 1 Department of Gynecology of Traditional Chinese Medicine, Affiliated Foshan Maternity & Child Healthcare Hospital, Southern Medical University (Foshan Maternity & Child Healthcare Hospital), Foshan, China; 2 Department of Gynecology, The First Affiliated Hospital of Guangzhou University of Chinese Medicine, The First Clinical Medical College, Guangzhou University of Chinese Medicine, Guangzhou, China; 3 Department of Neurosurgery, The Second Affiliated Hospital of Guangzhou University of Chinese Medicine, Guangzhou, China; 4 Anorectal Disease Institute of Shuguang Hospital, Shanghai, China; 5 Department of Anorectal Surgery, Shuguang Hospital, Shanghai University of Traditional Chinese Medicine, Shanghai, China; Chiang Mai University, THAILAND

## Abstract

**Introduction:**

Approximately 80% of children, adolescents and young adults (CAYA) cancer patients are expected to fully recover and survive for five years or more. The reproductive health is a particular area of their concern. Evidence demonstrates that previous therapeutic treatments for cancer and comorbidities may have harmful effects on female fertility and delivery outcomes, which will significantly affect patient quality of life. However, these reports are heterogeneous. Therefore, the purpose of this study is to provide the up-to-date evidence on the risk of adverse obstetric and perinatal outcomes in female survivors of childhood or adolescent cancer.

**Methods and analysis:**

This meta-analysis will be carried out and reported with adherence to the Meta-Analysis of Observational Studies in Epidemiology (MOOSE) guidelines and the Cochrane Handbook. We will search online databases including PubMed, Cochrane Library, and Embase from inception to August, 2022 to identify all relevant cohort studies examining the relationship between cancer and subsequent obstetric and perinatal outcomes. Data extraction from eligible studies will be conducted and crosschecked separately by two investigators using pre-tested standardized data extraction forms. Discrepancies will be resolved via a third investigator. Methodological quality assessment for each study will be conducted using the Newcastle–Ottawa scale (NOS) tool. We will apply the DerSimonian-Laird random-effects model to calculate the pooled estimates. Further sources of heterogeneity will be explored by performing subgroup analysis based on multiple study characteristics. Potential publication bias will be assessed by inspection of a funnel plot, Begg’s and Egger’s regression tests of funnel plot asymmetry.

**Ethics and dissemination:**

Ethical approval will not be required as all data used for this pooled analysis will be obtained from published cohort studies. The results of this study will be disseminated in a peer-reviewed journal and conference presentation.

**OSF registration number:**

DOI 10.17605/OSF.IO/K6QBG.

## Introduction

The increase in the number of children, adolescents and young adults (CAYA) cancer patients is becoming a growing concern, and such patients are expected to fully recover and survive for a long time [1–3]. The overall five-year survival rate of CAYA cancer patients is reported to be approximately 80% [4, 5]. As these survivors reach childbearing age, they will face the risk of physical and psychosocial complications caused by cancer itself and its treatment and reproductive health is a particular area of their concern [6]. There is often concern that their previous diseases and treatments may have harmful effects on female fertility and delivery outcomes, which will significantly affect their quality of life [7, 8].

Previous studies have found that cancer survivors have a higher risk of preterm birth and postpartum hemorrhage than cancer-free women, and that these risks are further inflated in patients who receive pelvic or abdominal radiotherapy [9–14]. Kaidar-Person et al. found that breast cancer survivors had an increased risk of acute obstetrical complications during delivery compared to other compared to other mothers [15]. In a recent systematic review, cancer survivors were found to be at increased risk for undergoing selective and emergency caesarean sections, as well as assisted vaginal delivery and postpartum haemorrhage [16]. However, Zgardau and colleagues did not find a correlation between abdominal or pelvic radiation and preterm delivery [17], which contradicts the previous studies that radiation impairs the growth of the pelvis and uterus, resulting in limited uterine volume or impaired vascular supply to the fetus, leading to preterm delivery [18, 19]. They also found no increased risk of preeclampsia, caesarean section or gestational diabetes among cancer survivors, which diverged from previous studies [17].

These contradictory results prompt us to conduct a comprehensive and systematic review with meta-analysis on the risk of adverse obstetric and perinatal outcomes in female survivors of childhood or adolescent cancer based on published high-quality population-based cohort studies.

## Methods and analysis

### Protocol registration

This meta-analysis will be reported and conducted based on the Meta-Analysis of Observational Studies in Epidemiology (MOOSE) guidelines and the Cochrane Handbook (http://handbook.cochrane.org) [20]. Its protocol will be reported with adherence to the Preferred Reporting Items for Systematic Reviews and Meta-Analyses Protocols (PRISMA-P) guidelines [21] (S1 Table). We have preregistered the protocol on the open science framework (OSF) website (https://osf.io/x2vra) with the registration number of DOI 10.17605/OSF.IO/K6QBG.

### Data sources and search strategies

We will search online databases (i.e., PubMed, Cochrane Library, and Embase) from inception to August, 2022 using the specific search strategies as presented in Table 1, in order to identify all relevant cohort studies examining the relationship between cancer and subsequent obstetric and perinatal outcomes. Both Medical Subject Heading (MeSH) /EMBASE Subject Headings (Emtree) terms combined with free text words that are associated with cancer, obstetric outcomes, perinatal outcomes, and cohort study will be used. The keywords will be used to identify relative studies: one including terms on cancer (cancer*, tumor*, tumour*, oncolog*, neoplasm*, malignanc*, carcinoma*), the other one regarding obstetric or perinatal outcomes (obstetric outcomes, obstetric complications, perinatal outcomes, perinatal complications), which will be linked with the Boolean operators AND or OR. We will also check clinical trial registration, reference lists of included studies, conference proceedings and relevant reviews to identify additional eligible studies. Manual search of abstracts from annual meetings of the American Gynecological and Obstetrical Society (AGOS), European Society of Gynecology (ESGO), the American Society of Clinical Oncology (ASCO) and the European Society for Medical Oncology (ESMO) will also be performed. When studies with duplicate data from the same cohort are published several times, only the most recent article with the largest population will be selected for inclusion. Search results will be imported into Endnote X7 (Thomson Reuters), where duplicate entries will be removed. Those additional duplicate entries will be manually removed by comparing authors, study titles and publication dates. Two authors will independently carry out the literature search, study selection, and the subsequent data extraction. Conflicts will be resolved by a senior author.

**Table 1:** Search strategy for pubmed database.

***Cancer Survivor terms:***
1 "Cancer Survivors"[MeSH]
2 (cancer* or oncolog* or neoplasm* or carcinom* or tumor* or tumour* or malignan* or hematooncological OR hemato oncological or hemato-oncological or hematologic neoplasms or hematolo*)[Title/Abstract]
3 (survivor* OR surviving) [Title/Abstract]
4 and/2-3
5 1 or 4
***Obstetric/Perinatal Outcome terms:***
6 "Premature Birth"[Mesh]
7 (preterm or premature) [Title/Abstract]
8 "Infant, Low Birth Weight"[Mesh]
9 birth weight[Title/Abstract]
10 "Perinatal Death"[Mesh]
11 "Perinatal Mortality"[Mesh]
12 (perinatal mortality)[Title/Abstract]
13 "Intensive Care, Neonatal"[Mesh]
14 (neonatal and intensive care) [Title/Abstract]
15 "Fertility"[Mesh]
16 fertil* [Title/Abstract]
17 conception [Title/Abstract]
18 "Pregnancy"[Mesh]
19 pregnancy[Title/Abstract]
20 gestation*[Title/Abstract]
21 "Abortion, Spontaneous"[Mesh]
22 miscarriage*[Title/Abstract]
23 "Cesarean Section"[Mesh]
24 (cesarean or caesarean) [Title/Abstract]
25 "Obstetric Labor, Premature"[Mesh]
26 "Labor, Obstetric"[Mesh]
27 (labor or labour) [Title/Abstract]
28 "Fetal Membranes, Premature Rupture"[Mesh]
29 pPROM[Title/Abstract]
30 "Hypertension, Pregnancy-Induced"[Mesh]
31 "Pre-Eclampsia"[Mesh]
32 (Hypertens* and Pregnan*) [Title/Abstract]
33 (Preeclamp* or Pre-eclamp* or eclamp*)[Title/Abstract]
34 "Diabetes, Gestational"[Mesh]
35 (gestational diabetes) [Title/Abstract]
36 (pregnancy induced diabetes) [Title/Abstract]
37 (pregnancy-induced diabetes) [Title/Abstract]
38 GDM[Title/Abstract]
39 "Placenta Previa"[Mesh]
40 (Placenta abruption)[Title/Abstract]
41 "Premature Birth"[Mesh]
42 Preterm[Title/Abstract]
43 "Postpartum Hemorrhage"[Mesh]
44 (post-partum haemorrhage or postpartum haemorrhage or post-partum hemorrhage) [Title/Abstract]
45 ((neonatal or perinatal or fetal or birth* or deliver*) and outcome*)[Title/Abstract]
46 "Stillbirth"[Mesh]
47 (stillborn or stillbirth) [Title/Abstract]
48 ((f?etal or intrauterine or intra-uterine) and (growth or death* or loss*))[Title/Abstract]
49 IUGR[Title/Abstract]
50 (small gestational age) [Title/Abstract]
51 (large gestational age) [Title/Abstract]
52 (Pregnancy complications) [Title/Abstract]
53 (Obstetric complications)[Title/Abstract]
54 (Pregnancy outcomes) [Title/Abstract]
55 (Birth outcomes) [Title/Abstract]
56 (Obstetric outcomes) [Title/Abstract]
57 (adverse outcomes) [Title/Abstract]
58 pregnanc*[Title/Abstract]
59 or/6-58
***Study design terms:***
60 "Retrospective Studies"[Mesh]
61 "Cohort Studies"[Mesh]
62 "Longitudinal Studies"[Mesh]
63 "Follow-Up Studies"[Mesh]
64 "Prospective Studies"[Mesh]
65 (cohort or longitudinal or followup or prospective*or retrospective* or database* or population* or follow up)[Title/Abstract]
66 "Registries"[Mesh]
67 (registry or registries) [Title/Abstract]
68 or/60-67
***Final search results: Combining Cancer and Obstetric/Perinatal Outcome and Study design:***
69 5 and 59 and 68

### Eligibility criteria

Studies with all inclusion criteria satisfying the PECOS framework will be included in the qualitative and quantitative analyses.

#### Study question

Are adverse obstetric and perinatal outcomes mostly due to cancer itself and cancer-related treatments for female survivors of childhood or adolescent cancer?

#### Study design

Prospective or retrospective population-based cohort studies published in any languages will be considered eligible;

#### Populations

This study will draw participants from female survivors of childhood or adolescent cancer diagnosed based on the International Classification of Diseases (ICD) criteria in the population-based cohort which reported one type of adverse obstetric and perinatal outcomes identified by ICD criteria;

#### Exposure

The primary exposure of interest was defined as cancer diagnosis and cancer-related treatment including chemotherapy, radiotherapy, or other related antitumor therapies.

#### Comparators

Studies will be included if they compared outcomes in the exposed group with those in a group of unexposed population (people who are cancer-free female controls).

#### Outcomes

Studies will be included if one of the primary outcomes including any adverse obstetric outcomes, obstetric complications, perinatal outcomes and perinatal complications are reported and their risk estimates are provided.

### Data extraction

Data extraction from eligible studies will be conducted and crosschecked separately by two investigators using pre-tested standardized data extraction forms. Discrepancies will be resolved via a third investigator. The following variables will be extracted from the included studies by applying a structured template: author of the publication, publication year, study set and design, research country and time range, participant mean or median age at cancer diagnosis, comparison population, criteria for ascertainment of cancer diagnosis and obstetric or perinatal outcomes, study results and risk estimates of the association of obstetric or perinatal outcomes with cancer.

### Critical appraisal and methodological quality assessment

Methodological quality assessment for each study will be conducted using the Newcastle–Ottawa scale (NOS) tool for cohort studies, with focus on the major aspects including participant selection, comparability of study groups, and ascertainment of exposure and outcomes [22]. The full score is nine and the high-quality study is defined as a study with quality scores being more than 7 and moderate to low quality as no more than 7 [23].

### Statistical analysis

We will carry out all statistical meta-analyses with the software Stata 15.0 (Stata Corp LP, College Station, TX). For each obstetric and perinatal outcome, the pooled relative risk (RR) in female cancer survivors compared with the RR in the cancer-free controls with its 95% CI will be estimated. To account for heterogeneity in the involved population, study setting or design, and outcome assessment, we will apply the DerSimonian-Laird random-effects model to calculate the pooled estimates [24]. We will use the fully adjusted RRs for the association between cancer and the outcomes to derive pooled estimates and present the results with forest plots. Because the incidence of adverse obstetric or perinatal outcomes was low, the hazard ratio (HR) from cohort studies is approximated to RR and they can be combined for meta-analysis. We will evaluate the between-study heterogeneity by applying the chi-square test on Cochran’s Q statistic, which is quantified by the I^2^ statistic, where an I^2^ > 50% representing significant heterogeneity [25]. We will further explore sources of heterogeneity by performing subgroup analysis when two or more studies for each subgroup are available for a given outcome according to the following variables: study setting and design, research region, methodological quality, age, adjuvant therapy used, cancer site and follow-up period. Meta-regression will be performed for eligible investigated outcomes to further explore potential sources of heterogeneity. Potential publication bias will be assessed by inspection of a funnel plot and Begg’s and Egger’s regression tests of funnel plot asymmetry if 10 or more studies are involved in the meta-analysis [26, 27]. Sensitivity analysis will be performed by removing each study at a time and reanalyzing the others to assess whether any individual study will significantly affect the pooled estimates. If results indicate possible publication bias, adjusted risk estimates will be calculated using the Duval and Tweedie nonparametric trim-and-fill technique [28].

## Discussion

To the best of our knowledge, this will be the first and most comprehensive meta-analysis to investigate the risk of adverse obstetric and perinatal outcomes in female survivors of childhood or adolescent cancer. This study will provide female cancer survivors with counseling and surveillance before and during pregnancy, and early risk warning for high-risk pregnant women who may develop adverse maternal outcomes, and offer evidence-based recommendations for those female survivors of childhood or adolescent cancer.

This study has several other strengths. Firstly, the Cochrane Handbook and PRISMA guideline will be strictly adhered to when conducting and reporting this meta-analysis and study quality of each included study will be assessed using NOS tool, which can objectively gain the quality of the pooled evidence. Secondly, by performing several subgroup analyses and sensitivity analyses, we can comprehensively explore the sources of between-study heterogeneity and confirm the robustness of the meta-analysis results. Finally, we will search the three major electronic databases and manually search other sources without language or date limitations to minimize the possibility of publication bias. Nevertheless, this study has a major limitation due to its anticipated significant between-study heterogeneity in that the included populations are female participants diagnosed with all types of cancer. However, this study is expected to reveal the relationship between cancer and obstetric and perinatal outcomes provide us a general risk estimate of obstetric and perinatal outcomes among female survivors of childhood or adolescent cancer.

## Supporting information

S1 Table PRISMA-P checklist. (DOC)

## Abbreviations

ASCO: the American Society of Clinical Oncology
AGOS: the American Gynecological and Obstetrical Society
CAYA: children, adolescents and young adults
Emtree: EMBASE Subject Headings
ESGO: the European Society of Gynecology
ESMO: the European Society for Medical Oncology
ICD: the International Classification of Diseases
MeSH: Medical Subject Heading
NOS: Newcastle Ottawa Scale
PRISMA-P: Preferred Reporting Items for Systematic Review and Meta-Analysis Protocols
RR: relative risk
